# Impact of infection with human immunodeficiency virus-1 (HIV) on the risk of cancer among children in Malawi - preliminary findings

**DOI:** 10.1186/1750-9378-5-5

**Published:** 2010-02-12

**Authors:** Nora Mutalima, Elizabeth M Molyneux, William T Johnston, Harold W Jaffe, Steve Kamiza, Eric Borgstein, Nyengo Mkandawire, George N Liomba, Mkume Batumba, Lucy M Carpenter, Robert Newton

**Affiliations:** 1 Epidemiology and Genetics Unit, Department of Health Sciences, Seebohm Rowntree Building, Area 3, University of York, York YO10 5DD, UK; 2 Department of Paediatrics, University of Malawi, College of Medicine, P/Bag 360 Chichiri, Blantyre 3, Malawi; 3 Department of Public Health, University of Oxford, Rosemary Rue Building, Old Road Campus, Roosevelt Drive, Headington, Oxford OX3 7LF, UK; 4 Department of Histopathology, University of Malawi, College of Medicine, P/Bag 360 Chichiri, Blantyre 3, Malawi; 5 Department of Surgery, University of Malawi, College of Medicine, P/Bag 360 Chichiri, Blantyre 3, Malawi; 6 Department of Ophthalmology, University of Malawi, College of Medicine, P/Bag 360 Chichiri, Blantyre 3, Malawi

## Abstract

**Background:**

The impact of infection with HIV on the risk of cancer in children is uncertain, particularly for those living in sub-Saharan Africa. In an ongoing study in a paediatric oncology centre in Malawi, children (aged ≤ 15 years) with known or suspected cancers are being recruited and tested for HIV and their mothers or carers interviewed. This study reports findings for children recruited between 2005 and 2008.

**Methods:**

Only children with a cancer diagnosis were included. Odds ratios (OR) for being HIV positive were estimated for each cancer type (with adjustment for age (<5 years, ≥ 5 years) and sex) using children with other cancers and non-malignant conditions as a comparison group (excluding the known HIV-associated cancers, Kaposi sarcoma and lymphomas, as well as children with other haematological malignancies or with confirmed non-cancer diagnoses).

**Results:**

Of the 586 children recruited, 541 (92%) met the inclusion criteria and 525 (97%) were tested for HIV. Overall HIV seroprevalence was 10%. Infection with HIV was associated with Kaposi sarcoma (29 cases; OR = 93.5, 95% CI 26.9 to 324.4) and with non-Burkitt, non-Hodgkin lymphoma (33 cases; OR = 4.4, 95% CI 1.1 to 17.9) but not with Burkitt lymphoma (269 cases; OR = 2.2, 95% CI 0.8 to 6.4).

**Conclusions:**

In this study, only Kaposi sarcoma and non-Burkitt, non-Hodgkin lymphoma were associated with HIV infection. The endemic form of Burkitt lymphoma, which is relatively frequent in Malawi, was not significantly associated with HIV. While the relatively small numbers of children with other cancers, together with possible limitations of diagnostic testing may limit our conclusions, the findings may suggest differences in the pathogenesis of HIV-related malignancies in different parts of the world.

## Background

People infected with HIV have been found to be at increased risk of developing certain cancers [1-5] including Kaposi sarcoma, non-Hodgkin lymphoma and conjunctival carcinoma [6]. To date, however, the majority of evidence has been based on studies of adults; the impact of infection with HIV on the risk of cancer in children is less certain. Studies in developed countries, have suggested approximately 2.5% of children infected with HIV will develop cancer, lower than the proportion seen among infected adults [3]. Cancers described as being associated with HIV in children include Kaposi sarcoma, non-Hodgkin lymphoma and leiomyosarcoma [3,7,8] but to date, there are few data from sub-Saharan Africa, where the majority of HIV infected children live. Here, we examine the association between HIV infection and cancer among children in Blantyre, Malawi.

## Results

Out of a total of 586 suspected cases of childhood cancer, 541 were recruited into the study. All children whose diagnosis was unknown were excluded (n = 27, 4.6%). Of the children included in the study, 284 (52%) were diagnosed with Burkitt lymphoma, 53 (10%) with nephroblastoma (Wilms tumour), 34 (6%) with Kaposi sarcoma, 36 (7%) with non-Burkitt non-Hodgkin lymphomas, 28 (5%) with rhabdomyosarcoma and 22 (4%) with retinoblastoma (Table 1). Children diagnosed with other types of cancer and non-malignant conditions comprised 15% of the children included in the analysis (84 children). There were no children diagnosed with leiomyosarcoma.

**Table 1:** General characteristics of 541 children with cancer diagnosed in Blantyre, Malawi

				Cancer diagnosis	n (%)				
	
	Burkitt lymphoma	Wilms tumour	Kaposi sarcoma	**Other lymphoma**^‡^	Rhabdo-myosarcoma	Retino-blastoma	**Other tumours**^¶^	**Non-malignant conditions**^†^	Total**
	284 (52)	53 (10)	34 (6)	36 (7)	28 (5)	22 (4)	61 (11)	23 (4)	541 (100)

***Sex:***									

Female	112 (39)	23 (43)	13 (39)	13 (36)	11 (39)	14 (64)	26 (43)	9 (39)	221 (41)

Male	172 (61)	30 (57)	20 (61)	23 (64)	17 (61)	8 (36)	35 (57)	14 (61)	319 (59)

Age at diagnosis*									

0 to 5 years old	57 (21)	37 (74)	12 (41)	5 (15)	14 (52)	20 (95)	21 (39)	10 (45)	176 (35)

≥ 5 years old	213 (79)	13 (26)	17 (59)	28 (85)	13 (48)	1 (5)	33 (61)	12 (55)	330 (65)

Mean (se)	7.47 (0.18)	4.09 (0.34)	7.24 (0.75)	9.40 (0.68)	5.43 (0.73)	3.10 (0.36)	7.25 (0.61)	5.68 (1.04)	6.91 (0.16)

***Residence***									

Rural	199 (71)	37 (73)	15 (45)	25 (69)	19 (68)	13 (65)	42 (70)	14 (64)	364 (69)

Urban	80 (29)	14 (27)	18 (55)	11 (31)	9 (32)	7 (35)	18 (30)	8 (36)	165 (31)

***Method of diagnosis***^∝^									

Clinical signs	38 (13)	8 (15)	18 (53)	7 (19)	8 (29)	7 (32)	8 (13)	7 (30)	101 (19)

Clinical investigations	46 (16)	5 (9)	11 (32)	9 (25)	6 (21)	5 (23)	8 (13)	5 (22)	95 (18)

Laboratory confirmed	197 (69)	37 (70)	5 (15)	20 (56)	13 (46)	10 (45)	43 (70)	11 (48)	336 (62)

Not Recorded	3 (1)	3 (6)	0 (0)	0 (0)	1 (4)	0 (0)	2 (3)	0 (0)	9 (2)

***HIV status***^¥^									

Positive	20 (7)	1 (2)	24 (77)	5 (14)	0 (0)	0 (0)	1 (2)	3 (14)	54 (10)

Negative	263 (93)	51 (98)	7 (23)	31 (86)	28 (100)	16 (100)	57 (98)	18 (86)	471 (90)

^‡^Non-Burkitt Non-Hodgkin lymphoma and unspecified lymphomas,^¶^Other tumours include: Neuroblastoma(17), Germ Cell tumours(16), Hepatic tumours(9), Bone tumours(5), Cranial tumours (4), Soft tissue sarcomas(4), Epithelial tumours (4), Renal carcinomas(1) and Other carcinomas (1), ^† ^Non-malignant conditions include: Aplastic anaemia, Benign cystic teratoma, Benign mesenchymal tumour, Cellular mesoblastic nephroma, Chronic inflammation, Chronic osteomyelitis, Dental cyst, Dentrigenous cyst, Encephalitis, Haemangioma, Liver abscess, Melanotic neuroectodermal tumour of infancy, Pleomorphic adenoma, Ranula, Salivary gland tumour, unclassified soft tissue tumour, Teratoma, Torted ectopic spleen and Tuberculosis, *35 patients had missing age data, ^∝ ^See methods section, ^¥^16 patients had missing HIV serology, **excludes 8 patients with leukaemia and 10 with Hodgkin lymphoma.

There was a preponderance of male children in the sample (59% overall) and most children (69%) resided in rural regions of the country (Table 1). The majority of the children were diagnosed after their fifth birthday (65%), a pattern observed for most of the cancer groupings, except for those with nephroblastoma or retinoblastoma; 35 children had missing age data. Overall, 62% of the cases had laboratory confirmed diagnoses and 37% had the diagnosis made on clinical grounds alone. Ten percent of all children (n = 54) tested positive for HIV, although HIV prevalence varied by cancer type, ranging from 0% among children with retinoblastoma and rhabdomyosarcoma to 77% (n = 24) among children with Kaposi sarcoma. Of the 506 children whose age was known, 25 children were under 18 months, and all were HIV seronegative.

HIV was strongly and positively associated with Kaposi sarcoma (OR = 93.5, 95% CI 26.9 to 324.4, *p *< 0.001) and positively associated with non-Burkitt non-Hodgkin lymphoma (OR = 4.4, 95% CI 1.1 to 17.9, *p *= 0.04), but not with Burkitt lymphoma (OR = 2.2, 95% CI 0.8 to 6.4, *p *= 0.13; Table 2). No other cancer site or type was significantly associated with HIV infection; although to date, the numbers available for analysis remain small. None of the 8 leukaemia cases and only 1 of the 10 cases of Hodgkin lymphoma were HIV seropositive. Analyses were repeated restricting to those cases with laboratory confirmation of diagnosis: key findings remain unchanged although confidence limits were wider (see footnote to Table 2).

**Table 2:** The association between HIV infection and specific cancer types^†^

**Adjusted prevalence of HIV infection (%)**^‡^			
	**Estimate**	95% CI*	
	
Burkitt lymphoma (n = 269)	9.1	(6.0 to 13.2)	
Kaposi sarcoma (n = 29)	81.8	(63.1 to 93.6)	
Non-Burkitt non-Hodgkin lymphoma (n = 33)	8.9	(1.8 to 24.0)	
All other cancers (n = 164)	3.5	(1.3 to 7.6)	

**Odds ratio for specific cancer given HIV infection compared to baseline^§ ^group**			

	**Odds ratio**	**95% CI**	***p*-value**
	
Burkitt lymphoma (n = 269)	2.2	(0.8 to 6.4)	0.132
Kaposi sarcoma (n = 29)	93.5	(26.9 to 324.4)	<0.001
Non-Burkitt non-Hodgkin lymphoma (n = 33)	4.4	(1.1-17.9)	0.038

^†^Individual logistic regression models of the risk of having a specific cancer based on HIV seroprevalence compared to the 'all other cancers' group were adjusted for age class and gender; patients diagnosed with nephroblastoma, retinoblastoma and rhabdomyosarcoma have been included in the all other cancers group; patients with missing age, gender or HIV status have been excluded. ^‡^Prevalence standardised to a population 50% female and 50% over 5 years old. *Exact binomial confidence limits. ^§^Baseline group includes 'all other cancers' and non-malignant conditions.

NOTE: When analyses were repeated restricting to those cases with a laboratory confirmation of diagnosis, the results materially similar: Burkitt lymphoma OR 2.0, 95% CI 0.5-7.7; Kaposi sarcoma OR 46.9, 95% 3.5-631.1; Non-Burkitt non-Hodgkin lymphoma OR 3.6, 95% CI 0.6-21.5.

## Discussion

Relative to adults, there are few published analytical epidemiological studies of cancer in HIV infected children, particularly among those living in sub-Saharan Africa, and the spectrum of cancers affecting children may be different [4]. Unlike adults, the great majority of HIV-infected children acquire the virus *in utero *or in the first months of life when the immune system is at an early stage of development. The proportion of children with HIV infection who will go on to develop a malignancy is at present poorly defined [3]. This study, carried out in Malawi, has found clear evidence of positive associations between HIV infection and both Kaposi sarcoma and non-Burkitt non-Hodgkin lymphoma in children. The relative risk of Burkitt lymphoma was raised, although not significantly.

While Kaposi sarcoma in Malawian children was relatively rare prior to the HIV epidemic [9], the number of cases has increased during the era of HIV [10-12]. In some sub-Saharan African countries, Kaposi sarcoma is now one of the most common cancers in children [13-20]. This increase is likely to reflect the prevalence of HIV, although it is also in an area with a high background prevalence of the underlying causal virus Kaposi's sarcoma-associated herpesvirus (KSHV) [21].

The increased risk for non-Burkitt non-Hodgkin lymphoma in association with HIV in Malawian children is of similar magnitude to that reported for adults in South Africa (OR = 5.0, 95% CI 2.7 to 9.5, based on 128 cases), Rwanda (OR = 12.6, 95% CI 2.2 to 54.4, based on 26 cases), and Uganda (OR = 6.2, 95% CI 1.9 to 19.9, based on 21 cases) [22-25]. In these studies, the relative risk of non-Hodgkin lymphoma associated with HIV infection is an order of magnitude lower than that reported from developed countries [1,26]. The reasons for this difference are not clear. Possible explanations for the apparent lack of non-Hodgkin lymphoma among HIV infected people in Africa include under-ascertainment of the malignancy and competitive mortality from other HIV-associated illnesses. As with all African series, a proportion of diagnoses are made on clinical grounds alone, and so we cannot completely exclude the possibility that some cases were misdiagnosed (e.g. tuberculosis or other infection-associated lymphadenopathy).

A case-control study from Uganda was the first to report an association between Burkitt lymphoma and infection with HIV among children living in an area where the tumour is relatively frequent [23] (note: a small subset of preliminary data from this study previously reported no association [27]). In 1994, no cases of Burkitt lymphoma were found in 78 HIV positive children in Côte d'Ivoire[28], and no increase in the incidence of childhood Burkitt lymphoma after the onset of the HIV epidemic was noted in Zambia [16]. A preliminary analysis of data from Malawi, published in early 2008, identified an excess risk of Burkitt lymphoma - based on 11 HIV infected cases and 228 without HIV (OR = 12.4, 95% CI 1.3 to 116.2)[29]. In the updated analyses reported here, the odds ratio is lower and no longer statistically significant, although the adjusted prevalence of HIV is similar to that of non-Burkitt non-Hodgkin lymphoma. However, the number of HIV positive children with Burkitt lymphoma reported in the literature to date is small and there remains substantial uncertainty about the role of HIV (if any) in the aetiology of this common malignancy among children in parts of sub-Saharan Africa. Our results and those of other African studies contrast with those from Western populations, where adults and children with AIDS are at least 1,000 times more likely to develop Burkitt-type lymphoma than the general population [1,23,30,31], probably reflecting different pathogenetic mechanisms.

The overall HIV seroprevalence of 10% found among children included in this analysis was as expected in a population of children admitted to hospital in Malawi [32], and is similar to that seen in children with cancer in Uganda [23]. For some cancers, the number of cases available at the time of writing was too small to draw reliable conclusions. Our results are broadly in line with those reported elsewhere in Africa [9,13,23,33-35], although data on cancer in HIV-infected children remains scant. In developed countries, both primary brain lymphomas and leiomyosarcomas tend to occur in children with HIV infection [3,36-40]. Brain tumours are difficult to diagnose in developing countries, although 4 cases with cranial tumours were reported here (all HIV seronegative). No cases of leiomyosarcoma were found.

Leiomyosarcoma is a very rare tumour, occurring in less than 2 cases per 10 million HIV negative children per year [41], although it occurs at a much higher than expected frequency in HIV infected children in developed countries [3,7,8,38]. There is paucity of data from African studies regarding the occurrence of leiomyosarcoma in HIV infected children. It is possible that leiomyosarcoma is under-diagnosed in this setting. Soft tissue tumours are diagnostically challenging, with histopathological criteria that are continuously changing. It is therefore possible that leiomyosarcomas may be diagnosed as rhabdomyosarcoma or other soft tissue tumours. Even in settings where more advanced tumour morphology, immunophenotyping and other ancillary investigations are done, discrepancies in diagnoses occur [42].

The results reported here are subject to the potential problems of incomplete diagnostic verification. Laboratory verification of cancer diagnosis by histology and cytology was available for 64% of all cancers. This proportion varied by cancer site; being higher if the tumour was easily accessible for biopsy. These results are typical of studies in developing countries, where laboratory services are limited, and compare favourably with other cancer series reported from Africa [13,14,43]. On the other hand, our study had the advantage of a high ascertainment of HIV status among all children with cancer (97%), which was consistently high across all cancer types, in contrast to a previous review of childhood cancer in Malawi [13]. It is possible, however, that children with both cancer and HIV were likely to die before a diagnosis could be made, potentially biasing our findings.

## Conclusions

The impact of HIV on the risk of cancers other than Kaposi sarcoma and non-Burkitt non-Hodgkin lymphoma remains uncertain and is the subject of further research. As data accrue from Malawi and elsewhere, the impact of HIV on the risk of other cancer types among children should be clarified.

## Methods

### Recruitment and data collection

All children aged 15 years or younger with a provisional diagnosis of cancer admitted to the paediatric wards at Queen Elizabeth Hospital in Blantyre, Malawi, are recruited into a study of childhood cancer. Here we present findings for children recruited between July 2005 and March 2008. Children with eye malignancies are cared for on a separate mixed adult and children's ophthalmology ward, and recruitment for the ophthalmology patients was only carried out between July 2005 and July 2006. Preliminary clinical diagnoses of cancer were made by one investigator (EMM) and confirmed by histology, cytology or other laboratory investigations where possible. Based on available information, all diagnoses were coded to the International Classification of Childhood Cancer, 3^rd ^edition [44]. Five local nurses were employed and trained to recruit children and their mothers into this study and to administer standardized questionnaires. The parent or guardian of each child was approached and invited to participate in the study and provide written informed consent for their child to be included. All children seen in the paediatric oncology ward with suspected cancer were routinely tested for HIV infection using Determine HIV (Abbott Laboratories, Illinois, USA) as a screening test and Uni-Gold™ HIV (Trinity Biotech PLC, Ireland) as a confirmatory test, as used in other studies from Malawi [13,29,45]. Cancer diagnosis was established using varying methods. In some instances diagnosis was based on clinical symptoms and signs only. Clinical investigations included X-rays, ultrasound, blood counts and peripheral smears. Where possible, cancer diagnoses were laboratory confirmed, meaning the diagnoses were histologically verified either by cytology or histology of tumour tissue. Appropriately trained staff provided pre- and post-HIV test counselling. Ethical approval for the study was obtained from the Oxford Tropical Research Ethics Committee and the Malawian College of Medicine Research and Ethics Committee.

### Statistical analyses

Initial descriptive analyses retained groupings of cancer types with at least 20 children diagnosed. Cancer types with fewer than 20 cases were included in a single group called 'other tumours' (illustrated in Table 1). All children diagnosed with non-Burkitt non-Hodgkin lymphomas (and unspecified lymphomas) were included in a single group called 'other lymphomas' (shown in Table 1). For assessing the risk of having a specific cancer associated with HIV infection, children diagnosed with nephroblastoma, retinoblastoma and rhabdomyosarcoma were included with the smaller diagnostic groups due to small numbers of HIV positive children with these specific cancer diagnoses (an aggregate group called 'all other cancers', as illustrated in Table 2). The 'all other cancers' group (n = 164) and non-malignant diagnoses (n = 23) were grouped together to form a 'control' or 'baseline" group (n = 187) of which 164 children had complete age, sex and HIV status data. A series of unconditional logistic regression models of the risk of each cancer group (Burkitt lymphoma, Kaposi sarcoma and non-Burkitt, non-Hodgkin lymphomas) being associated with HIV infection was constructed by maximum likelihood, adjusting for the child's age at diagnosis (under 5 years, and 5 years and over) and sex: the 'baseline' group was used as the controls for each logistic regression. The association between HIV status and the risk of being diagnosed with one of the other cancer types with more than 20 patients (nephroblastoma, rhabdomyosarcoma or retinoblastoma) was assessed by removing patients with the diagnosis of interest from the baseline group and comparing them to those patients remaining. Kaposi sarcoma and lymphomas, which are known to be associated with HIV, were excluded from the comparison group, as were all other haematological malignancies (leukaemia n = 8, Hodgkin lymphoma n = 10 and small round blue cell tumours n = 4 (a descriptive diagnosis that might include any of the following tumours: neuroblastoma, rhabdomyosarcoma, non-Hodgkin lymphoma, Ewing's sarcoma, primitive neuroectodermal tumour and the blastemic component of nephroblastoma [46])) because of possible diagnostic overlap with lymphomas [47-49].

## List of abbreviations

HIV: (Human Immunodeficiency Virus); AIDS: (Acquired Immunodeficiency Syndrome); OR: (Odds ratio); CI: (Confidence Interval); KSHV: (Kaposi's sarcoma-associated herpesvirus).

## Competing interests

The authors declare that they have no competing interests.

## Authors' contributions

NM, RN, EMM and LMC conceived of the study, and participated in its design and coordination and helped to draft the manuscript. WTJ performed the statistical analysis. SK, EB, NM, GNL, MB and HWJ participated in drafting the manuscript. All authors read and approved the final manuscript.
